# Uptake of HIV and syphilis testing of pregnant women and their male partners in a programme for prevention of mother-to-child HIV transmission in Uganda

**DOI:** 10.1111/j.1365-3156.2008.02052.x

**Published:** 2008-05

**Authors:** Dennison Kizito, Patrick W Woodburn, Beleth Kesande, Christine Ameke, Juliet Nabulime, Moses Muwanga, Heiner Grosskurth, Alison M Elliott

**Affiliations:** 1 Medical Research Council/Uganda Virus Research Institute (MRC/UVRI) Uganda Research Unit on AIDSEntebbe, Uganda; 2 Entebbe HospitalEntebbe, Uganda; 3 London School of Hygiene and Tropical MedicineLondon, UK

**Keywords:** HIV, PMTCT, Uganda, pregnant, couple, syphilis

## Abstract

**Objective:**

To describe uptake of HIV and syphilis testing in a prevention of mother-to-child HIV transmission programme in Uganda.

**Methods:**

Analysis of data from routine HIV and syphilis testing at Entebbe Hospital antenatal services.

**Results:**

A total of 20 738 women attended antenatal services. Exactly 62.8% of women, but only 1.8% of their male partners, accepted testing for HIV; 82.2% of women, but only 1.1% of their male partners accepted syphilis testing. Partners of women with positive HIV results were more likely to come for subsequent testing. Of 200 couples whose partners accepted HIV-testing within 30 days of one another, 19 (9.5%) were HIV-discordant, representing 65.5% of couples with at least one partner HIV-positive. HIV prevalence was 12.6% for women and 10.8% for men; syphilis prevalence was 4.0% for women and 6.2% for men.

**Conclusion:**

Uptake of HIV and syphilis testing was fairly good among pregnant women attending antenatal clinics at Entebbe Hospital, but very low among their male partners. The level of HIV-discordant couples was high. These clinics should be made more couples-friendly to identify both HIV-positive men for treatment and discordant couples for HIV prevention.

## Introduction

HIV testing in antenatal clinics can support interventions that address maternal health and prevention of mother-to-child HIV transmission (PMTCT), but uptake of opt-in testing (where tests are offered, as opposed to opt-out, where they are done routinely) is sometimes low in resource-poor settings (Medley *et al.* 2004; Mpairwe *et al.* 2005; Karamagi *et al.* 2006) with low rates of HIV serostatus disclosure to their partners being reported among women tested in antenatal services. This has implications for prevention of transmission between serodiscordant partners (Painter 2001) and for PMTCT programmes (Medley *et al.* 2004): adherence to interventions is difficult for women whose partners are unaware, or not supportive of, their participation (Farquhar *et al.* 2004). Thus, promoting involvement of male partners in PMTCT programmes may improve uptake, behaviour change and compliance to PMTCT measures (such as medication with nevirapine and choices for infant feeding) (Gaillard *et al.* 2002;The Republic of Uganda Ministry of Health (MoH) 2004) and it may allow appropriate counselling and intervention for discordant couples.

We examined the uptake of HIV counselling and testing by pregnant women and their male partners at an antenatal clinic in Entebbe, Uganda, and compared this with the uptake of syphilis testing. We also examined the effect of the woman's HIV status on whether the male attended later (if he had not attended the first visit) and the frequency of serodiscordance within couples.

## Methods

In keeping with the policy of the Ugandan Ministry of Health (The Republic of Uganda Ministry of Health MoH) 2004), a PMTCT programme using nevirapine was initiated in 2002 at Entebbe Hospital (a district referral hospital in a semi-urban setting). All women receiving antenatal services are registered and receive health education and group counselling about syphilis, HIV infection, PMTCT and the occurrence of HIV discordance within couples. These women are offered individual counselling before blood samples are drawn for testing at an on-site laboratory providing same-day results. HIV-testing is performed only if the test is accepted, whereas syphilis testing is routine. Once tested, women proceed for medical examination while the samples are processed. Post-test counselling (including advice on HIV prevention) is given when they receive their results. Women with positive syphilis serology are treated with benzathine penicillin according to the MoH guidelines. Syphilis-positive and HIV-positive women are advised to invite their partners to visit the clinic for counselling, testing and medical care if required. More recently, all women have been encouraged to do so, particularly with regard to HIV-testing. HIV-positive women are given support counselling and advised about infant-feeding options. They are asked to return at 28 weeks of pregnancy to collect nevirapine tablets to be taken at the onset of labour. If they are diagnosed as HIV-positive at 32 weeks or later, tablets are provided on the same day. Within 72 h after delivery, women are advised to bring their babies to receive nevirapine syrup.

HIV tests were performed using a serial testing algorithm (triple rapid testing). The particular tests used have changed due to changes in availability over time (Branson 2000; Respess *et al.* 2001). Syphilis serology is performed using a rapid plasma reagin (RPR) test: any positive result is regarded as sero-syphilis and the woman treated accordingly. Assay materials are supplied by the MoH. Quality control is provided through the Medical Research Council/Uganda Virus Research Institute (MRC/UVRI) Uganda Research Unit on AIDS and has shown excellent results consistently.

Our analysis was carried out on data held on HIV and syphilis status and uptake by pregnant women and their partners attending the antenatal clinic. Data on the socioeconomic or demographic characteristics of these individuals are not held.

## Results

From May 2002 to January 2006, 20 738 pregnant women registered at the antenatal clinic; 17 293 (83.4%) accepted individual counselling, of whom 13 029 (75.3%) accepted an HIV test while 17 056 (98.6%) were tested for syphilis. The reasons why some women did not accept counselling or testing have not been systematically explored. Among those who accepted testing, results are available for 12 978 HIV tests and 16 985 syphilis tests. Only 236 male partners attended for HIV and syphilis counselling, of whom 191 (80.9%) requested both syphilis and HIV tests, 41 (17.4%) requested HIV testing only and 4 (1.7%) syphilis testing only. Among the couples tested, the HIV prevalence was 12.6% [95% confidence interval (CI) 12.1–13.2%] for women and 10.8% (7.1–15.5%) for men; syphilis prevalence was 4.0% (3.7–4.3%) for women and 6.2% (3.3–10.6%) for men.

To determine the effect of a woman's HIV status on the probability of her partner subsequently attending, we considered 57 couples where the male partner did not attend the first visit. We excluded 167 couples who were tested on the same date because the decision to attend by the male partner may not have been informed by knowledge of his partner's status, and eight where only the male partner accepted testing. We compared the proportion of women whose partner later reported for an HIV test to the proportion whose partner never reported (Table 1). We found that partners of HIV-positive women were twice as likely to seek knowledge of their own HIV status.

**Table 1:** The proportion of pregnant women whose male partners later requested an HIV test by woman's HIV status

	Partner later requested an HIV test?		
	
Woman's HIV status	Yes	No	χ^2^*P*-value
Positive	13 (0.8%)	1614	0.022
Negative	44 (0.4%)	11 129	

We examined the related question of whether the time until a male partner reported for testing was affected by the woman's HIV status, but found no difference (mean time if female partner was HIV-negative, 52.5 days; mean time if female partner was HIV-positive, 45.4 days; Kruskal–Wallis test, *P* = 0.80).

Specimens for HIV-testing were collected either on the same day or, for 200 couples, within 30 days for both partners. Nineteen (9.5%; 95% CI 5.8–14.4%) of these were HIV-discordant: nine female-positive, male-negative; ten female-negative, male-positive. Ten couples (5.0%; 2.4–9.0%) were concordant positive, 171 (85.5%; 79.8–90.1%) concordant negative. Among couples with at least one partner HIV-positive, the rate of discordance was 19/29 (65.5%; 45.7–82.1%), an important level of HIV discordance.

## Discussion

This analysis of an antenatal clinic in Uganda has observed a very low level of male partner HIV-testing and syphilis-testing, and a high level of HIV discordance among couples who do accept testing.

Our data highlight the challenge of promoting couple HIV-testing within a PMTCT programme, because only 1.8% of women tested had partners who accepted an HIV test. It is possible some male partners not seen at Entebbe Hospital may have gone to alternative centres (e.g. to an AIDS support NGO), to private clinics in the same community, or to various centres in Kampala (40 km away), and that others may already have been aware of their status. We do not think that this has been the case for many of the men concerned. The population of this area has generally very low incomes and prefers the free public services offered within the community; while using the services of AIDS support NGOs is often still associated with the fear of stigma and is avoided by men who perceive themselves to be in good health.

The observation that more male partners of HIV-positive women attended, while ostensibly encouraging, can plausibly be ascribed to counselling bias, because HIV-positive women were specifically advised to bring their partners. We have started to explore whether men make better use of testing services, if these services are offered not within the antenatal clinic but through a separate ‘male-friendly, couple-friendly’ clinic, and initial results are encouraging (data not shown). We look forward to seeing how this compares with the effect of switching to opt-out testing for both parents, as carried out in Tororo, Eastern Uganda (Homsy *et al.* 2006), and to the sending of postal invitations to partners, as carried out in Mwanza, Tanzania (Abdallah *et al.* 2006).

Although our comparatively small sample of couples may not be representative for all couples with at least one HIV-positive partner in Entebbe, the high proportion of serodiscordance observed clearly argues for interventions to reduce HIV transmission in stable marital partnerships. Antenatal services offer important potential for HIV prevention if more male partners could be attracted to attend. The fact that discordance occurred equally in both directions emphasises the importance of couple-testing for all participants, not just for HIV-positive women; and in our clinic, this policy has replaced the earlier approach, which emphasised couple testing for women identified as HIV-positive.
